# Unpacking the relationship leader affiliative humor and employee innovative behavior: multilevel roles of role breadth self-efficacy and team trust

**DOI:** 10.3389/fpsyg.2025.1629241

**Published:** 2025-10-10

**Authors:** Zheli Zhang, Zhenting Xu, Ruixiao Yu

**Affiliations:** ^1^Personnel Department, Zhejiang Tongji Vocational College of Science and Technology, Hangzhou, China; ^2^Business School, Qingdao University of Technology, Qingdao, China; ^3^School of Logistics, Linyi University, Linyi, China

**Keywords:** employee innovative behavior, leader affiliative humor, role breadth self-efficacy, team trust, multilevel research

## Abstract

Employee innovative behavior stands as a pivotal factor influencing the core competitiveness of enterprises. Extant research indicates that leadership style is a crucial factor driving employee innovative behavior. Based on social information processing theory, this study focuses on the multilevel mechanism of leader affiliative humor (LAFH) on employee innovative behavior. Through a multi-source, two-stage questionnaire survey, paired sample data were collected from 79 leaders and 302 employees. The results indicate that LAFH has a significant positive effect on role breadth self-efficacy, team trust, and employee innovative behavior, respectively. Role breadth self-efficacy and team trust play a multilevel mediating role in the relationship between LAFH and employee innovative behavior. Besides, team trust exerts a significant multilevel moderating effect on the relationship between LAFH and role breadth self-efficacy, with a stronger positive relationship when team trust is higher.

## 1 Introduction

Innovation serves as the driving force for long-term business development and is essential for maintaining their sustainable competitive advantage (Zhang et al., 2022; Lohani and Blodgett, 2025). As frontline personnel across various roles, employees are well-positioned to provide valuable insights and suggestions that reflect the fundamental problems existing within the organization. Their creativity fuels innovation by offering fresh perspectives that improve products and work methods. Employee innovative behavior refers to the process wherein employees generate, introduce, and apply useful and novel ideas or procedures in organizational activities (Awan and Jehanzeb, 2022). In practice, organizational leaders continuously take proactive measures to enhance employee innovative behavior, thereby meeting the constantly changing environment (Elsetouhi et al., 2023). Based on this, identifying the key factors that influence employee innovative behavior has been the focal point of attention in both theoretical and practical domains.

Scholarly inquiry on the influencing factors of employee innovative behavior has made progress, for instance, perceived innovation job requirement, high-involvement work practices, team reflexivity, mobile workplace stress, perceived overqualification, etc. (Wang et al., 2022; Ma et al., 2023). Of note, researchers believe that leaders significantly affect employee innovative behavior, in that, leaders, as key figures within organizations, allocate scarce resources and encourage participation in innovative activities through rewards, recognition, and promotion opportunities (Ye et al., 2022). Furthermore, leadership theories suggest that leaders influence employee innovative behavior on one hand by affecting individual-level factors such as attitudes, abilities, and cognition, while on the other hand, they create a team climate via interactions with subordinates or by role modeling, thereby exerting an impact on employee innovative behavior (Xu et al., 2022). However, compared to other prominent leader attributes and behaviors (e.g., leader trait positive affect, extraversion, and consideration behaviors), leader humor has demonstrated a greater impact on employees’ behaviors and performance, for instance, organizational citizenship behavior and job performance (Cooper et al., 2018; Kong et al., 2019). Likewise, the extant literature has demonstrated that leader humor, serving as a social lubricant, can improve employees’ attitude and cognition by enhancing communication between leaders and employees (Cooper et al., 2018), may influence employee innovative behavior. Based on this, this study aims to explore the relationship between leader humor and employee innovative behavior.

Although progress has been made, important limitations remain. First, knowledge about the relationship between leader humor and employee innovative behavior is still limited. For instance, Pundt (2023) has revealed that humor in leadership affects employee innovative behavior. However, in accordance with Martin et al.'s (2003) typology, leader humor can be classified into four categories: leader affiliative humor, aggressive humor, self-defeating humor, and self-enhancing humor. Leader affiliative humor refers to a manager’s use of jokes, humorous anecdotes, and witty remarks to elicit laughter from employees, thereby fostering interpersonal connections with subordinates. Leader aggressive humor encompasses sarcasm, teasing, mockery, and belittling others, often by ridiculing their mistakes. Self-enhancing humor represents a positive form of self-directed humor that involves maintaining a humorous perspective when confronting stressful events and adversities. The latter refers to excessively demeaning or mocking oneself to gain approval from others. Compared with self-defeating humor and self-enhancing humor (viewed as self-directed leader humor styles), leader affiliative humor and aggressive humor, as self-directed leader humor styles, are most relevant for understanding the “push and pull” dynamics of leaders’ use of humor on employees’ cognition and behavior at work (Cooper et al., 2018). Meanwhile, the extant literature has found that leader aggressive humor exerts a negative effect on employee innovative behavior (Diastari and Parahyanti, 2023). Based on this, this study focuses on leader affiliative humor (LAFH), which enhances the relationship between leaders and their team members, and explores its relationship with employee innovative behavior. Particularly, the extant literature has ignored the multilevel attribution of LAFH, and its multilevel effect on employee innovative behavior.

Second, limited understanding exists regarding the mechanisms through which LAFH affects employee innovative behavior. According to social information processing (SIP) theory, subordinates perceive and process information about the behaviors exhibited by their leaders (social cue), subsequently influencing their subsequent attitudes and behaviors (Salancik and Pfeffer, 1978). Guided by SIP theory, LAFH, on the one hand, through sharing interesting anecdotes and engaging in friendly interactions, can be easily perceived by subordinates as a sign of leaders’ benevolence and amicable demeanor (Xu et al., 2023). On the other hand, through humorous communication, leaders implicitly convey values (e.g., “innovation is encouraged”, “mistakes are acceptable”), which reduces fear of making errors. This, in turn, enhances employees’ task confidence, raises their role breadth self-efficacy, eventually improving employee innovative behavior. Likewise, LAFH, through engaging in friendly interactions, can create a relaxed and enjoyable climate within the organization (Yang and Wen, 2021; Xu et al., 2023), thereby cultivating team trust, eventually enhancing employee innovative behavior. Hence, this study will adopt role breadth self-efficacy and team trust as mediating role to unpack the mechanism by which LAFH affects employee innovative behavior.

Finally, SIP theory posits that social contexts, including task characters or team climate, shape how individuals interpret leaders’ social cues (Salancik and Pfeffer, 1978). Team trust, defined as the shared belief that team members can rely on and have confidence in one another, fosters a positive and supportive team climate (Edmondson, 1999). By facilitating communication, cooperation, and coordination (Breuer et al., 2016), team trust can influence how employees process leader affiliative humor (LAFH) and form perceptions such as role breadth self-efficacy. Based on this, this study investigates the moderating role of team trust in the relationship between LAFH and role breadth self-efficacy.

Overall, our study makes three theoretical contributions to the existing literatures. First, our study responds to the recent call from humor research for future research should offer fresh theoretical insights into how followers perceive and respond to leader humor (Cooper et al., 2018; Kong et al., 2019). Meanwhile, it expands the existing literature on humor by going beyond the multilevel attribution of LAFH to examine its impact on employee innovative behavior. Second, our study uncovers the mechanism through which LAFH affects employee innovative behavior, as well as enriching the theoretical explanation of SIP theory in the relationship between LAFH and employee innovative behavior. Finally, by introducing team trust as a moderator, our study unpacks the boundary condition in the relationship between LAFH and employee innovative behavior.

## 2 Literature and hypotheses development

### 2.1 LAFH and employee innovative behavior

Leader humor is conceptualized from two perspectives: the behavioral perspective and the trait perspective. From a behavioral perspective, Avolio et al. (1999) define leader humor as the extent to which leaders employ positive humor in stressful situations. While Cooper et al. (2018) describe it as an intentional leader behavior aimed at eliciting laughter, relieving stress, and fostering positive interpersonal relationships with employees. The trait perspective treats humor as an individual characteristic that varies in terms of its creation, understanding, and approach. Martin et al. (2003) category leader humor into four dimensions: affiliative, aggressive, self-enhancing, and self-deprecating. Among them, LAFH refers to leaders using jokes and humorous stories to make employees laugh. Research on LAFH has shown its positive influence on employee work engagement, extra-role behaviors, and job performance (Rosenberg et al., 2021; Xu et al., 2023).

SIP theory posits that the influence of leaders on their subordinates is contingent upon how subordinates perceive and process social cues from leader behavior (Salancik and Pfeffer, 1978). LAFH, characterized as a benign and non-hostile form of humor, entails narrating amusing stories, sharing jokes, and crafting well-intentioned jests to entertain others (Martin et al., 2003). From the lens of employees, as recipients of leader humor, they view leaders as more approachable and open, which encourages interactions between employees and leaders, such as the exchange of thoughts and suggestions concerning ongoing tasks, thereby facilitating employee innovative behavior.

Besides, LAFH can be considered a mild form of norm violation (Yam et al., 2018). According to SIP theory, such signals prompt employees to challenge established routines, thereby sparking employee innovative behavior. Meanwhile, the extant literature has demonstrated that leaders’ use of humor to share novel ideas, thereby improving employee innovative behavior (Pundt, 2023). Hence, we hypothesize the following:

*H1*: LAFH is positively related to employee innovative behavior.

### 2.2 Mediating role of role breadth self-efficacy

Self-efficacy denotes individuals’ belief in their ability to utilize their skills to accomplish tasks, enabling employees to pursue work goals and promoting proactive behavior (Bandura, 1978; Cattelino et al., 2023). Role breadth self-efficacy refers to employees’ belief in their capability to perform a broader range of tasks that exceed formal job requirements and demand higher-level skills (Kang et al., 2022). Compared with self-efficacy, role breadth self-efficacy encompasses a wider range of responsibilities, thereby facilitating employees to engage in extra-role behaviors and undertake innovative tasks through generating new ideas and introducing novel work procedures (Ngo et al., 2023). Besides, the existing studies have revealed that high performance work systems, learning goal orientation, perceived overqualification, and leader support are positively associated with role breadth self-efficacy (Ngo et al., 2023).

According to SIP theory, leaders play a pivotal role as social information sources, and shape employees’ attitudes and behaviors by conveying cues that employees interpret cognitively (Salancik and Pfeffer, 1978). Based on this, LAFH signals warmth, approachability, and benevolence, thereby strengthening leader–member exchange and emotional rapport (Cooper et al., 2018). These interactions foster positive affect and reduce perceived threat, increasing employees’ confidence in handling broader and more challenging tasks. Meanwhile, the existing literature has demonstrated that leaders, by employing affiliative humor, delight subordinates and instill in them positive emotions (Yam et al., 2018). By doing so, subordinates become filled with confidence and courage, thereby enhancing their role breadth self-efficacy (Kang et al., 2022). Therefore, LAFH has a positive impact on role breadth self-efficacy.

Besides, role breadth self-efficacy, a key driver of individual behavior, profoundly shapes cognition, affect, and action (Hwang et al., 2015). Expectancy theory posits that individuals prefer behaviors aligning with their expectations (Behling and Starke, 1973). Employees with high role breadth self-efficacy are more likely to believe in their ability to manage situations and evaluate their actions’ effectiveness. When such employees also perceive that new ideas benefit the organization, they are more motivated to initiate innovative activities. Moreover, employees with high role breadth self-efficacy also demonstrate greater initiative and foresight, prompting engagement in novel tasks (Markman et al., 2002). Meanwhile, the extant studies have revealed that role breadth self-efficacy plays an important role in employee innovative behavior (Odoardi, 2015). As aforementioned above, we argue that LAFH positively affects via role breadth self-efficacy. Hence, we hypothesize the following:

*H2*: Role breadth self-efficacy mediates the relationship between LAFH and employee innovative behavior.

### 2.3 Mediating role of team trust

Trust is an individual’s psychological state characterized by positive expectations about others’ intentions or actions, enabling individuals to disclose weaknesses without fear of exploitation (Tomlinson et al., 2020). Team trust is a collective attribute, reflecting positive expectations among team members regarding each other’s behavior (Zheng and Wang, 2023). It is also the belief that team members make sincere efforts in accordance with explicit and implicit commitments (Legood et al., 2023). Moreover, the extant literature has noted that team trust contributes to team cohesion and team performance (Zheng and Wang, 2023).

According to SIP theory, employees encode and store workplace cues—such as leaders’ behaviors—which shape their cognitive judgments and subsequent actions (Salancik and Pfeffer, 1978). Guided by SIP theory, when leaders exhibit affiliative humor and employees perceive it, they interpret this behavior as a signal of friendliness and an effort to foster a positive communication climate within the organization (Kong et al., 2019). This elicits positive emotions, strengthens leader–member ties, and increases interaction among team members, thereby building team trust. Moreover, Cooper et al. (2018) has demonstrated that leader humor can form a high-quality relationship between leaders and employees and enhances employees’ sense of support and trust. Therefore, LAFH can cultivate team trust.

Besides, team trust can shape employees’ collective perception of the workplace (Legood et al., 2023). When employees believe they will not be exploited for revealing weaknesses, they are more willing to invest time and effort in creative activities aimed at improving products or services. With the support of colleagues or leaders, they collaboratively facilitate the conversion of ideas into creative outcomes, thereby enhancing employee innovative behavior (Zhao et al., 2023). Meanwhile, social exchange theory posits that trust serves as the foundational basis for social exchange (Cropanzano and Mitchell, 2005). Based on this, the heightened level of trust among employees correlates with an increased sense of duty and responsibility, thereby prompting individuals to display altruistic behaviors surpassing their role expectations, contributing to the welfare of their team (Xu et al., 2022). This objectively promotes a sense of duty among employees to enhance the quality of organizational products or services and facilitates the generation of innovative ideas. In addition, the extant literature has demonstrated that team trust has a positive impact on innovative behavior (Kmieciak, 2021; Xu et al., 2022). Guided by SIP theory, LAFH, considered as social cue, is perceived and interpretated friendliness by employees, thereby shaping positive team climate (team trust), subsequently improving employee innovative behavior. As aforementioned above, we argue that LAFH has a positive impact on employee innovative behavior via team trust. Hence, we hypothesize the following:

*H3*: Team trust mediates the relationship between LAFH and employee innovative behavior.

### 2.4 Moderating role of team trust

SIP theory suggests that the information processing of individuals is shaped not only by direct social cues but also by team and task characteristics (Salancik and Pfeffer, 1978). Besides, Huang and Luthans (2015) further argue that understanding individual behavior requires attention to interactions between individuals and their external environment. Notably, team trust reflects the positive expectations among team members concerning their interpersonal relationships and serves as an evaluation of the prevailing trust climate within the team (Burke et al., 2007). Consistent with SIP, this process requires individuals to continuously collect and interpret relevant information as a basis for judging the trustworthiness of team members (Salancik and Pfeffer, 1978). Based on SIP theory, this study posits that team trust moderates the relationship between LAFH and role breadth self-efficacy.

The connection between LAFH and role breadth self-efficacy can be viewed as leaders signaling support and trust, which boosts employee confidence and consequently strengthens role breadth self-efficacy (Cooper et al., 2018; Kong et al., 2019). The extant literature has revealed that team trust not only has a direct impact on team performance but also fosters a climate of mutual assistance and support, facilitating communication and collaboration among team members (De Jong et al., 2016). Therefore, for teams with higher levels of team trust, employees are inclined to interpret the social information conveyed by LAFH in a more positive manner, namely, they believe that LAFH denotes friendliness and support. This encourages employees to focus on the positive facets of LAFH, enhancing their sense of responsibility and confidence, thus increasing their role breadth self-efficacy. Conversely, for teams with lower levels of team trust, employees are inclined to interpret the social information conveyed by LAFH as negative information, perceiving their work environment as unfriendly. This, in turn, strengthens employees’ perception of threat and hostility within the work environment, dampening their confidence and sense of responsibility, ultimately reducing their role breadth self-efficacy. Based on this, we hypothesize the following:

*H4*: Team trust moderates the relationship between LAFH and role breadth self-efficacy, that is, the higher the team trust, the stronger the positive relationship between LAFH and role breadth self-efficacy.

**Figure 1:**
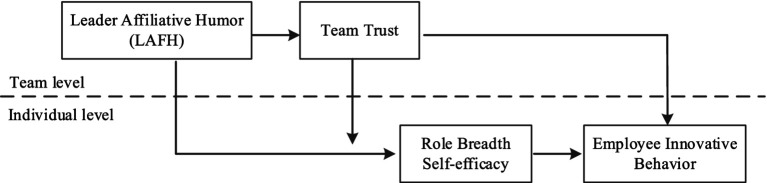
Conceptual framework.

The conceptual framework of this study is depicted in Figure 1.

## 3 Methods

### 3.1 Participants and procedure

This study sampled leaders and employees from small and medium-sized enterprises across Shanghai, Chongqing, Beijing, Shenzhen, and other regions, covering various industries, including manufacturing, services, and finance. The surveyed companies attach great importance to the role of innovation in their organizational development. To reduce common method bias, this study adopted a time-lagged approach. Before the formal survey, we recruited two research assistants who formed a dedicated team responsible for the distribution and collection of questionnaires. Afterward, the research team provided a comprehensive explanation of the study’s objectives and procedures to the leaders of the surveyed companies, ensuring the exclusive use of questionnaires for academic research while maintaining strict confidentiality. With leaders’ permission, the research team organized the email information of both surveyed leaders and employees. They compiled a research information table based on the leader-subordinate pairing, sending out corresponding questionnaires at each stage and meticulously recording the delivery and retrieval times of the surveys.

To mitigate common method bias, this study adopted the approach of Podsakoff and Organ (1986) and strictly employed a two-wave design for data collection with a three-month interval. (1) The first survey was conducted from April to June 2024. It involved employees completing questionnaires on LAFH, team trust, and role breadth self-efficacy. Additionally, demographic variables such as gender, age, and education level were collected for the subordinates. A total of 529 questionnaires were distributed, and 416 returned. (2) Three months later, the second survey was conducted from September to October 2024 and involved leaders evaluating the innovative behavior of their subordinates. In this phase, 416 questionnaires were distributed and 320 returned. After removing invalid responses and unmatched pairs, the research team ultimately obtained 79 sets of paired questionnaires from leaders and 302 from subordinates, yielding an effective questionnaire recovery rate of 57.09%.

In the sample of managers: in terms of gender, males account for 63.3%, while females account for 36.7%; in terms of age, those aged 25–35 account for 38.0%, 36–45 account for 45.6%, and 46–60 account for 16.4%; when considering education level, the majority hold a bachelor’s degree, accounting for 60.8%, while those with a master’s degree or higher account for 31.6%; in terms of hierarchical positions, grassroots managers account for 54.4%, mid-level managers account for 34.2%, and senior managers account for 11.4%.

Within the sample of employees, the gender distribution reveals that 59.6% are male and 40.4% are female; in terms of age, those aged below 25 account for 16.5%, those aged between 25 and 35 account for 40.1%, those aged between 36 and 45 account for 33.8%, and those between 46 and 60 account for 9.6%; regarding educational level, 22.2% have an educational level below a bachelor’s degree, 55.3% hold a bachelor’s degree, and 22.5% hold a master’s degree or higher.

### 3.2 Measurement

All measurement scales employed in this study are sourced from established scales. To enhance the cross-cultural adaptability of the adopted scales, we used a back-translation procedure: one management expert translated the scales into Chinese, a second expert back-translated them into English, and a bilingual management scholar reviewed both versions for consistency. Moreover, we conducted a pilot study incorporating key procedures such as item analysis, reliability tests, and exploratory factor analysis (EFA), which facilitated the identification and comprehension of measurement biases arising from cultural differences, thereby ensuring construct validity across cultures.

#### 3.2.1 LAFH

LAFH was assessed by adopting the 8-items scale developed by Martin et al. (2003). One of the sample questionnaire items was “My supervisor usually does not laugh or joke around much with other people”®. The value of Cronbach’s alpha was 0.877.

#### 3.2.2 Role breadth self-efficacy

Guided by Strauss et al. (2009), this study assessed employees’ role breadth self-efficacy by three items with highest factor loadings in the scale developed by Parker (1998). The sample questionnaire items were “How confident you would feel carrying out new tasks,” “How confident you would feel contacting people outside,” and “How confident you would feel analyzing a long-term problem to find a solution.” In addition, we conducted confirmatory factor analysis (CFA) via AMOS 22.0 statistical analysis software. The results indicate a good fit with GFI = 0.941 NFI = 0.96, CFI = 0.977, SEA = 0.063. The response scale ranged from 1 (not at all confident) to 5 (very confident). The value of Cronbach’s alpha was 0.867.

#### 3.2.3 Team trust

Team trust was measured using a five-item scale developed by De Jong et al. (2016). An example item was: “When I encounter difficulties, I trust that my colleagues will help me solve them.” The value of Cronbach’s alpha was 0.889.

#### 3.2.4 Employee innovative behavior

Employee innovative behavior was measured using a six-item scale developed by Scott and Bruce (1994). An example item was: “In our team, my employees demonstrate full support for novel ideas.” The value of Cronbach’s alpha was 0.851.

#### 3.2.5 Control variable

Following prior studies (Wang et al., 2022; Ma et al., 2023), we included control variables to account for their potential effects on employee innovative behavior. At the individual level, controls included demographics such as gender and education; at the group level, controls included leaders’ age and position. Moreover, key constructs, such as LAFH, role breadth self-efficacy, team trust, and innovative behavior, were assessed using a Likert 5-point scale, with the exception of the control variables. Meanwhile, the value of Cronbach’s alpha of the aforementioned scales all exceeded the recommended value of 0.70, signifying high measurement quality in this survey.

## 4 Results

### 4.1 Confirmatory factor analysis

This study conducted confirmatory factor analysis (CFA) via AMOS 22.0 statistical analysis software. The hypothesized four-factor model (Model 1: LAFH, role breadth self-efficacy, team trust, and innovative behavior) was compared with other alternative nested models (Models 2–5), as detailed in Table 1. The results indicate a good fit for the hypothesized four-factor model, with *χ^2^*/df = 2.600, GFI = 0.907, NFI = 0.910, CFI = 0.918, RMSEA = 0.063. These values meet the criteria for statistical validation, with χ^2^/df values between 1 and 5, and CFI, GFI, NFI exceeding 0.9, and RMSEA below 0.08 (Hu and Bentler, 1999). Clearly, the hypothesized four-factor model demonstrates higher goodness of fit compared to the other alternative nested models (Models 2–5), indicating better discriminant validity.

**Table 1:** Confirmatory factor analysis (CFA).

Model	*χ^2^/df* (df)	GFI	CFI	NFI	RMSEA
Model 1: LAFH; RBSE; TT; EIB	2.600 (203)	0.907	0.918	0.910	0.063
Model 2: LAFH+RBSE; TT; EIB	3.889 (206)	0.803	0.831	0.786	0.098
Model 3: LAFH; RBSE+TT; EIB	4.227 (206)	0.777	0.811	0.767	0.104
Model 4: LAFH+RBSE+TT; EIB	5.866 (208)	0.673	0.712	0.674	0.127
Model 5: LAFH+RBSE+TT + EIB	7.197 (209)	0.625	0.631	0.598	0.143

LAFH denotes leader affiliative humor; RBSE denotes role breadth self-efficacy; TT denotes team trust; EIB denotes employee innovative behavior; “+” denotes the combination of variables.

### 4.2 Common method bias

Data for LAFH, role breadth self-efficacy, and team trust were collected from employees. Thus, to assess common method bias, we performed Harman’s single-factor test. The results revealed that the variance explained by the first factor before rotation was 22.67%, which is below the recommended threshold of 50%. Besides, through collinearity analysis, it was observed that the variance inflation factors (VIF) for all variables were below 2, and the tolerance exceeded 0.5, indicating no severe multicollinearity.

### 4.3 Team level data aggregation

We conducted tests on r_wg_ and interclass variability (ICC) to support the data aggregation of team-level variables, specifically LAFH and team trust. It is worth highlighting that LAFH refers to team leaders using jokes and humorous stories to make employees laugh. Thus, this study considered LAFH as team-level variable. Statistical calculations revealed the r_wg_ for LAFH is 0.87, with ICC (1) at 0.19 and ICC (2) at 0.80. For team trust, the r_wg_ is 0.85, with ICC (1) at 0.18 and ICC (2) at 0.78. All these values meet the statistical aggregation standards, i.e., r_wg_ > 0.7, ICC (1) > 0.12, ICC (2) > 0.7 (James et al., 1993). Therefore, both LAFH and team trust exhibit strong internal consistency and significant intergroup variability, allowing for the aggregation of scores for these two variables at the team level.

### 4.4 Descriptive statistics and correlation analysis

Using SPSS 22.0 statistical analysis software, this study analyzed the mean, standard deviation, and correlation of each variable, as shown in Table 2. From Table 2, it can be observed that at the individual level, role breadth self-efficacy is significantly positively correlated with employee innovative behavior (*r* = 0.39, *p* < 0.01). Furthermore, at the team level, LAFH is also significantly positively correlated with team trust (*r* = 0.61, *p* < 0.01), providing preliminary evidence for subsequent hypothesis testing.

**Table 2:** Mean, standard deviation and correlation analysis.

Variable	Mean	SD	1	2	3
Individual level					
Gender	1.31	0.46			
Educational level	3.30	0.73	0.06		
RBSE	3.80	0.62	−0.01	0.01	
EIB	3.93	0.61	0.02	0.03	0.39**
Team level					
Manager’s age	3.05	0.57			
Position	2.18	0.61	0.07		
LAFH	4.01	0.37	0.04	0.07	
TT	4.34	0.42	0.02	0.03	0.61**

**p < 0.01.

### 4.5 Hypothesis testing

To test the multilevel mediating effects of role breadth self-efficacy and team trust, along with the multilevel moderating effect of team trust, hierarchical linear model (HLM 6.08) was used in this study, and the results were shown in Table 3. Table 3 revealed a positive predictive impact of LAFH on employee innovative behavior (M2: *γ*_01_ = 0.40, *p* < 0.01). Thus, Hypothesis 1 was supported. LAFH significantly predicted role breadth self-efficacy (M6: *γ*_01_ = 0.64, *p* < 0.01). Moreover, there existed a significant correlation between role breadth self-efficacy and employee innovative behavior (Table 2: *r* = 0.39, *p* < 0.01). These findings aligned with the three hypotheses proposed by Mathieu and Taylor (2007) for validating multilevel mediation conditions. When LAFH and role breadth self-efficacy jointly explicated employee innovative behavior, the coefficient of LAFH on employee innovation significantly decreased (M2: *γ*_01_ = 0.60, *p* < 0.01 → M3: *γ*_01_ = 0.40, *p* < 0.01). Consequently, LAFH affects employee innovative behavior via role breadth self-efficacy. Meanwhile, this study adopted the Monte Carlo method for assessing the robustness of the mediating role of role breadth self-efficacy, the results showed that the effect of LAFH on employee innovative behavior through role breadth self-efficacy was 0.0925, and its confidence interval at 95% level was [0.0316 0.1735], excluding 0. Thus, Hypothesis 2 was supported.

**Table 3:** The results of multilevel effect of role breadth self-efficacy and team trust.

Variable	EIC				RBSE			
	M1	M2	M3	M4	M5	M6	M7	M8
Intercept (*γ_00_*)	3.92** (0.12)	3.66** (0.11)	3.03** (0.10)	3.62** (0.10)	3.80** (0.12)	3.21** (0.10)	2.79**	1.98**
Gender	0.05 (0.08)	0.04 (0.06)	−0.03 (0.10)	0.02 (0.08)	0.06 (0.05)	0.04 (0.04)	0.03 (0.03)	0.02 (0.02)
Educational level	0.09 (0.11)	0.06 (0.09)	0.04 (0.06)	0.04 (0.05)	0.05 (0.07)	0.04 (0.06)	0.02 (0.05)	0.02 (0.05)
Manager’s age	0.02 (0.04)	0.02 (0.03)	0.04 (0.05)	0.02 (0.04)	0.03 (0.05)	0.01 (0.03)	0.01 (0.03)	0.01 (0.03)
Position	0.04 (0.06)	0.02 (0.05)	0.03 (0.06)	0.01 (0.04)	0.02 (0.04)	0.01 (0.04)	0.02 (0.03)	0.01 (0.03)
LAFH(*γ_01_*)		0.60** (0.07)	0.40** (0.13)	0.46** (0.12)		0.64** (0.10)	0.45** (0.08)	0.23^**^ (0.09)
KSSE(*γ_10_*)			0.23** (0.08)					
TT(*γ_02_*)				0.16** (0.06)			0.26** (0.08)	0.20^**^ (0.06)
LAFH*TT(*γ_11_*)								0.04^*^ (0.02)
*σ^2^*	0.24	0.25	0.20	0.26	0.25	0.25	0.23	0.15
*τ_00_*	0.13	0.04	0.12	0.11	0.14	0.07	0.12	0.17

*p < 0.05, **p < 0.01; σ^2^ is the residual of level 1, τ_00_ is the intercept residual of level 2, the numbers in parentheses represent the standard errors.

Likewise, this study repeated the same mediation testing procedure aforementioned. From Table 3, it can be observed that LAFH had a significantly positive predictive effect on employee innovative behavior (M2: *γ*_01_ = 0.40, *p* < 0.01). Second, LAFH had a significant positive correlation with team trust (Table 2: *r* = 0.61, *p* < 0.01). Furthermore, team trust positively affects innovative behavior (M4: *γ*_02_ = 0.16, *p* < 0.01). The aforementioned conditions satisfied the three hypotheses proposed by Mathieu and Taylor (2007) for validating multilevel mediation conditions. When simultaneously explaining employee innovative behavior with LAFH and team trust, the coefficient of LAFH on employee innovative behavior significantly decreased (M2: *γ*_01_ = 0.60, *p* < 0.01 → M4: *γ*_01_ = 0.46, *p* < 0.01). Meanwhile, this study adopted the Monte Carlo method for assessing the robustness of the mediating role of team trust, the results showed that the effect of LAFH on employee innovative behavior through team trust was 0.0737, and its confidence interval at 95% level was [0.0259 0.1919], excluding 0. Thus, Hypothesis 3 was supported.

To investigate the multilevel moderating impact of team trust, we constructed a multilevel linear model to investigate the impact of the interaction between LAFH and team trust on role breadth self-efficacy. Initially, a baseline model was established (M5). Subsequently, LAFH was integrated into the baseline model to assess its influence on role breadth self-efficacy (M6). Furthermore, the effect of team trust on employee role breadth self-efficacy was analyzed post the inclusion of LAFH (M7). Finally, the interaction between LAFH and team trust on the influence of role breadth self-efficacy was tested (M8). The results were presented in Table 3. From Table 3, it can be observed that team trust positively moderated the relationship between LAFH and role breadth self-efficacy (M8, γ_11_ = 0.04, *p* < 0.01). Thus, Hypothesis 4 was supported.

**Figure 2:**
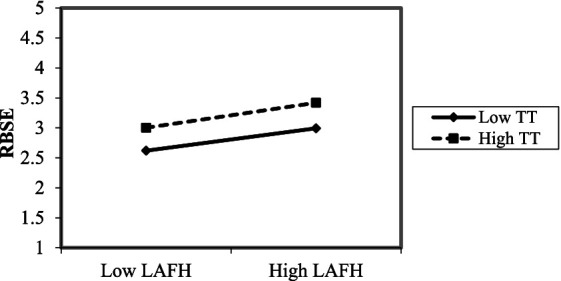
The interactive impact of and LAFH and team trust on role breadth self-efficacy.

Moreover, following the moderation approach recommended by Aiken et al. (1991), the interactive impact of LAFH and team trust on role breadth self-efficacy is depicted in Figure 2. From Figure 2, it can be observed that the positive relationship between LAFH and role breadth self-efficacy is stronger at higher levels of team trust than at lower levels.

## 5 Discussions

Drawing on SIP theory, using paired sample data of 79 leaders and 302 employees as the research sample, this study investigates the multilevel impact of LAFH on employee innovative behavior, focusing on the roles of role breadth self-efficacy and team trust in their relationship. The findings revealed: (1) LAFH exerted a significant and positive multilevel influence on employee innovative behavior; (2) role breadth self-efficacy and team trust play a multilevel mediating role in the relationship between LAFH and employee innovative behavior; (3) team trust exhibited a multilevel moderating effect on the relationship between LAFH and role breadth self-efficacy, wherein higher team trust strengthens this positive association.

First, the rapid development of the knowledge economy poses a primary challenge for leaders: how to promote employee innovative behavior to sustain and enhance organizational competitive advantage. Existing research attempts to explain the relationship between leadership behavior and employee innovative behavior, the mechanisms, situational characteristics. It suggests that leaders’ characteristics and behaviors (e.g., leader trait positive affect, entrepreneurial leadership, etc.) stand as significant predictive factors for employee innovative behavior, while neglecting the research on the relationship between LAFH and employee innovative behavior (Cooper et al., 2018). LAFH, on one hand, shares commonalities with other leadership behaviors (charismatic leadership, transformational leadership, servant leadership, etc.) and influencing employee behavior. On the other hand, in terms of leader-employee relationships, alternate leader behaviors primarily amplify employees’ intrinsic motivation to increase their proactiveness, thereby yielding benefits for organizations and employees. Conversely, LAFH, as a positive socioemotional resource offered by the leader, signals support and amicability, strengthens employees’ confidence, and promotes employees’ proactive and extra-role behaviors (Kong et al., 2019). This, in turn, leads to facilitating the generation and implementation of new ideas, thereby enhancing employee innovative behavior. Furthermore, LAFH possesses multilevel attributes, yet most studies examine leadership at a single level and neglect team-level effects on employee attitudes and actions. Therefore, this study focuses on the multilevel attributes of LAFH, exploring its multilevel relationship with employee innovative behavior. The research results also indicate that LAFH contributes to promoting employee innovative behavior.

Second, prior research holds that leaders influence employee innovative behavior in two ways: by shaping individual systems (cognition, capability, motivation) and by acting as “atmosphere engineers” who shape team climate (Xu et al., 2023). Based on this, integrating SIP theory with existing research conclusions, this study believes that LAFH signals support and warmth to employees, which is conducive to boosting employees’ confidence and hope, increasing their role breadth self-efficacy, and thereby enhancing employee innovative behavior. Furthermore, as an ‘atmosphere engineer’, leader will create a positive team climate that affects employee behavior. Therefore, LAFH can also influence employee behavior by creating a positive team climate, as LAFH benefits mutual trust. Meanwhile, the positive team climate (team trust, etc.) created by LAFH will further encourage employees to undertake creative or challenging tasks, thereby promoting their innovative behavior. Thus, based on SIP theory, this study explores how LAFH positively influences employee innovative behavior through role breadth self-efficacy (employees’ system) and team trust (team climate). Empirical results show that LAFH not only has positive effects on team trust and role breadth self-efficacy, respectively, but also positively influences employee innovative behavior via role breadth self-efficacy and team trust.

Finally, team trust shapes team members’ shared perception of their environment, promoting communication and collaboration within the team, and thereby impacting employee behavior. Based on this, this study treats team trust as a contextual moderator in the theoretical model and examines its moderating effect on the relationship between LAFH and role breadth self-efficacy. The results reveal that team trust significantly strengthens the positive relationship between LAFH and role breadth self-efficacy. A High team trust fosters a positive team climate and encourages leader–member communication and collaboration, which increases employees’ confidence and willingness to take on broader roles, thereby stimulating role breadth self-efficacy. Conversely, the inverse holds true. These results support prior work indicating that interactions between team context and individual-level factors influence employee behavior and outcomes, enhancing the contextual characteristics of their relationship.

### 5.1 Theoretical implications

Based on SIP theory, this study explores the multilevel relationship between LAFH and employee innovative behavior, with a particular focus on the effects of role breadth self-efficacy and team trust in their relationship. The main theoretical contributions are reflected in the following aspects.

First, research on leadership and innovation widely acknowledges leadership as a key determinant of employee innovative behavior. Leaders exerts its impact on employee innovative behavior through the integration of employee systems (cognition, capability, motivation, etc.) and team climate. Based on this, our study investigates whether and how LAFH influences employee innovative behavior. The results indicate that LAFH has a multilevel positive impact on employee innovative behavior. Therefore, this study contributes to enriching our systematical understanding of the multilevel attribution of LAFH and its positive effect on employee innovative behavior, thereby further expanding the literature on leader humor.

SIP theory posits that the work environment of team members, including leadership behavior, serves as a critical source of information, which shapes employees’ cognition and behavior (Salancik and Pfeffer, 1978). This study explores the multilevel mediating effects of role breadth self-efficacy (employee system) and team trust (team climate) to investigate their roles in the relationship between LAFH and employee innovative behavior. The results validate a dual mediating pathway of role breadth self-efficacy and team trust between LAFH and employee innovative behavior. Specifically, LAFH positively influences employee innovative behavior by affecting individual-level role breadth self-efficacy and nurturing a team climate of team trust. These findings contribute to unveiling the significant theoretical implications of SIP theory in the process of LAFH influencing employee innovative behavior, and clarifying the multilevel mechanisms through which LAFH impacts employee innovative behavior.

Third, studies on innovation posit that the examination of employee innovative behavior should not solely focus on individual-level factors (individual characteristics, capabilities, etc.), but should also incorporate the situational factors in which individuals are situated, clarifying the contextual characteristics through which individual factors influence employee innovative behavior. The results confirm that team trust significantly strengthens the positive relationship between LAFH and role breadth self-efficacy. This contributes to clarifying the boundary conditions of how LAFH influences role breadth self-efficacy and, to a certain extent, enriches the research on team trust, further enhancing the contextual characteristics of how leader humor influences employees’ cognition.

### 5.2 Practical implications

Employee innovative behavior has become a crucial source and driving force for the survival and development of organizations. This study finds that LAFH has a positive impact on employee innovative behavior. Therefore, it is imperative for enterprises to take practical steps to cultivate LAFH, such as training interventions or leader development programs. Leaders on the one hand can cultivate LAFH by means of training interventions or leader skill development program, on the other hand, can shape a positive and inclusive climate for enhancing communication and cooperation among employees. Meanwhile, leaders ought to prioritize honing their own sense of humor, taking into account various factors such as choosing appropriate scenarios and employing different humorous languages for employees with different psychological states.

Besides, the results indicate that LAFH exerts a positive impact on employee innovative behavior via role breadth self-efficacy. Thus, organizations should prioritize bolstering employees’ role breadth self-efficacy. Based on this, during the initial recruitment phases, enterprises should target candidates displaying resilience and confidence. Afterward, emphasis should provide sufficient resources (training programs, resource allocation, time, etc.) to subordinates, enabling them to receive assistance in problem identification, information search, and other crucial aspects. This, in turn, helps them build and enhance confidence, thereby boosting role breadth self-efficacy.

In the relationship between LAFH and role breadth self-efficacy, team trust plays a significant multilevel positive moderating effect on their relationship. Put differently, in a team with high level of team trust, the positive impact of LAFH on role breadth self-efficacy becomes more pronounced. Therefore, organizations can foster a positive team trust atmosphere by building sincere, mutual-trust, affirmative, and equal internal communication networks or platforms, cultivating a well-rounded, harmonious, and healthy corporate culture, and strengthening communication and collaboration between leaders and their employees.

### 5.3 Limitations and future directions

The limitations of this study are primarily evident in the following aspects: (1) Given the multilevel research design, this study cannot explore the causal relationship between LAFH and employee innovative behavior. Future research could employ longitudinal or experimental designs to clarify the causal relationships among variables in this study. Meanwhile, this study identified certain demographic variables at the individual level (such as gender, education level, etc.) and the group level (manager’s age, position) as control variables. However, control variables (such as team size, organizational type, industry characteristics, and team tenure) potentially impacted the relationship between LAFH and employee innovative behavior. Thus, future research could incorporate these control variables to uncover their potential impact on the relationship between LAFH and employee innovative behavior. (2) Except for employee innovative behavior, which leaders evaluated, the other measures (LAFH, role breadth self-efficacy, and team trust) were self-reported by employees, potentially introducing common-method bias. Hence, future studies should obtain measures from multiple sources—employees, peers, and supervisors—or use objective indicators and temporal separation to minimize this bias. (3) This study mainly focuses on individual and team levels, without considering the influence of organizational-level variables on the research model. Therefore, future research could incorporate organizational-level variables such as corporate culture and executive characteristics into the research model, comprehensively considering the systematic impact of various factors on innovative behavior. (4) This study views team trust as a moderator in the relationship between LAFH and role breadth self-efficacy. However, we conduct our study in the Chinese context, particularly, neglect how high-power-distance culture may affect employees’ interpretation of leader humor. Thus, future research should incorporate reflections on cultural factors and explore how these factors influence employees’ interpretation of leader humor, subsequently affecting individual cognition and behavior.

## Data Availability

The raw data supporting the conclusions of this article will be made available by the authors, without undue reservation.
